# Parental Death and Psychiatric Disorders Among Individuals with and Without Experience of Out-of-Home Care: A Swedish Nationwide Cohort Study

**DOI:** 10.3390/ijerph23060732

**Published:** 2026-05-30

**Authors:** Süheyla Seker, Lars Brännström, Hilma Forsman, Sandra Rogne

**Affiliations:** 1 Department of Social Work, Stockholm University, 106 91 Stockholm, Sweden; hilma.forsman@socarb.su.se; 2 Department of Criminology, Stockholm University, 106 91 Stockholm, Sweden; lars.brannstrom@criminology.su.se; 3 Department of Public Health Sciences, Stockholm University, 106 91 Stockholm, Sweden; sandra.rogne@su.se; 4 Centre for Health Equity Studies (CHESS), Stockholm University/Karolinska Institute, 106 91 Stockholm, Sweden

**Keywords:** parental death, psychiatric disorders, longitudinal study, Cox regression analysis, child welfare, out-of-home care

## Abstract

**Highlights:**

**Public health relevance—How does this work relate to a public health issue?**

- Children and adolescents in out-of-home care experience higher rates of psychiatric disorders and early death of one or both parents compared to non-placed peers.
- Placed children and adolescents are a highly vulnerable population due to the accumulation of psychosocial and familial disadvantages, making them relevant for public health investigation.

**Public health significance—Why is this work of significance to public health?**

- Placed individuals, particularly those first placed in adolescence, showed an increased risk for psychiatric disorders in adulthood, and parental death was also associated with an elevated psychiatric risk, highlighting the considerable public health burden of this population.
- Associations between parental death and psychiatric disorders were attenuated for individuals with experience of out-of-home care.

**Public health implications—What are the key implications or messages for practitioners, policy makers and/or researchers in public health?**

- Findings suggest that parental death adds limited risk within an already highly vulnerable population, emphasizing the already high disadvantage among individuals with experience of out-of-home care.
- This study highlights the importance of interventions addressing multiple risks across developmental periods for care and health systems and public health practitioners.

**Abstract:**

Out-of-home care and parental death are both associated with psychiatric disorders. It remains unclear whether parental death moderates the association between out-of-home care and psychiatric disorders, particularly regarding timing and duration of out-of-home placement. This cohort study included 944,138 individuals (459,712 women and 484,426 men) born in Sweden (1972–1981). Of these, 23,106 individuals (2.5%) experienced out-of-home care between ages 0 and 19. Psychiatric disorders, identified from the National Patient Register, were examined as the outcome. Information on parental death between ages 0 and 19 years and familial covariates was obtained from linked national registers. Sex-stratified Cox proportional hazard models examined the associations between out-of-home care, parental death, and psychiatric disorders from ages 20 to 47 years. Individuals with experience of out-of-home care had increased psychiatric risk across adulthood, with the highest risks observed for teenage placements. Parental death was also associated with an increased psychiatric risk. Associations between parental death and psychiatric disorders were attenuated across the teenage, long-term, and early intermediate placement groups among women and across the early intermediate, teenage, and early short-term placement groups among men compared with non-placed counterparts. Findings suggest that parental death adds limited risk within a highly vulnerable and disadvantaged population, highlighting the importance of interventions addressing multiple risks across developmental periods.

## 1. Introduction

Children and adolescents who experience severe familial adversities, such as maltreatment, are removed from their parental homes to ensure their safety [1]. The early loss of one or both parents can be a traumatic experience [2,3] and can be one of the reasons for out-of-home placement of the child [4], increasing the risk of subsequent mental health issues. In Sweden, the context of the present study, approximately 1% of children and adolescents enter out-of-home care annually due to maltreatment or risks to their physical or psychological development [5,6], and an estimated 3–4% of children and adolescents experience out-of-home care at some point during childhood or adolescence [7,8]. The Swedish child welfare system is family-oriented and aims for family reunification of the placed child with their biological parents whenever possible [6]. Accordingly, maintaining contact with biological parents is generally considered a substantial intervention during out-of-home care [8]. The mental health of individuals with experience of out-of-home care is a major public health concern, as this population is disproportionately exposed to early psychosocial and familial adversities across childhood and adolescence [9,10].

Although both out-of-home care and parental death have been independently associated with elevated psychiatric risks, it remains unclear whether parental death further compounds psychiatric vulnerability among children already exposed to substantial adversity, as indicated by placement, or whether its additional impact may be attenuated in highly disadvantaged populations. This unresolved question reflects two competing life-course perspectives. These perspectives generate distinct hypotheses regarding how multiple adversities may jointly influence psychiatric risk across the life course. First, the accumulation-of-disadvantage model proposes that disadvantages and adversities accumulate over the life course [11]. From this perspective, exposure to both parental death and out-of-home care would be expected to exert additive or compounding effects on psychiatric risk. Accordingly, individuals exposed to both adversities would be expected to show particularly elevated risks of psychiatric disorders compared to either adversity alone. Second, the disadvantage-saturation model hypothesizes that the level of disadvantage could reach a point of saturation, at which highly burdened individuals are less affected by experiencing additional strains and adversities in terms of opportunities for the future [12]. From this perspective, the additional impact of parental death may be attenuated among individuals with experience of out-of-home care who are already exposed to extensive psychosocial adversity. Accordingly, parental death would be expected to confer comparatively smaller additional psychiatric risks to individuals with experience of out-of-home care.

Research in developmental psychopathology has investigated how exposure to risk factors is associated with psychiatric vulnerability [13]. Children and adolescents in out-of-home care show substantially elevated rates of psychiatric disorders compared to the general population. Almost half of children and adolescents in out-of-home care meet the criteria for at least one mental disorder [14], with rates about three times higher than in the general population [15]. A meta-analytic review revealed that almost 30% of adults with a history of out-of-home placement had a mental disorder, with significantly higher odds for having a mental disorder compared to the general adult population [16]. Previous research thus highlights the vulnerability to adult psychiatric disorders among individuals with experience of out-of-home care [17,18,19,20].

Parental death during childhood and adolescence has likewise been identified as one of the most distressing life events [21] and has been associated with a range of poor psychosocial outcomes, such as self-inflicted injuries [22] and psychiatric disorders [23,24,25,26,27]. In Sweden, approximately 4–5% of all children experience the death of a parent before their 18th birthday [28], with similar rates reported in other Western countries [29,30]. Among individuals with experience of out-of-home care, rates of parental death are substantially higher [31]. A Swedish study reported that over 25% of adults with experience of out-of-home care had lost at least one parent before the age of 18 years, compared to 3–4% in the general population [32]. Parental death has been associated with a longer duration in care and a lower likelihood of reunification [4]. Additionally, the removal of a child from their parent per se can cause severe emotional distress and mental health problems, particularly for mothers [33], and lead to an increased risk of parental death by suicide [34]. These findings suggest that parental death may represent an additional adversity contributing to psychiatric risks among already disadvantaged children and adolescents.

At the same time, findings from previous research also suggest that the additional impact of parental death can be less pronounced among individuals with experience of out-of-home care. A recent Swedish cohort study found that parental death was not associated with an increased risk of premature mortality among out-of-home placed individuals compared with non-placed counterparts, and parental death did not further increase mortality risks beyond those associated with placement alone [31]. Continued contact with biological parents may also be emotionally complex for children in care and may contribute to distress, conflicting loyalties, and attachment-related difficulties [35,36]. These findings may therefore be more consistent with the disadvantage-saturation perspective, suggesting that additional adversities such as parental death may confer limited additional risk in populations already characterized by substantial psychosocial burden.

Developmental timing may also be important when considering these theoretical perspectives. Exposure to adversity during different developmental stages may shape attachment processes, emotional regulation, and psychiatric vulnerability differently across the life course. For example, placements occurring during adolescence often reflect distinct developmental pathways characterized by behavioral dysregulation, substance misuse, or delinquency [7], whereas childhood placements may more often reflect chronic family dysfunction and prolonged exposure to neglect or maltreatment. To date, however, it remains unclear how the timing and duration of placement, as well as the accumulation of familial adversities, contribute differently to long-term psychiatric risks.

In summary, previous studies have largely focused on the main effects of out-of-home care and parental death on adult outcomes. Yet, a longitudinal study examining the relationship between parental death and adult psychiatric disorders among out-of-home placed children and adolescents, a vulnerable population marked by high levels of disadvantage, is lacking. Moreover, no study has examined whether parental death modifies the association between timing and duration of care and adult psychiatric disorders. In this context, Cox proportional hazard regression analyses can provide insight into how early familial and environmental adversities are associated with psychiatric outcomes over time. Therefore, the aims of the present prospective cohort study, using Swedish register-based data from approximately 1,000,000 individuals, were threefold: (a) to investigate the association of out-of-home care with psychiatric disorders, (b) to examine the relationship of parental death with psychiatric disorders, and (c) to explore how parental death moderates the relationship between out-of-home care and adult psychiatric disorders.

To our knowledge, this is the first cohort study to investigate whether parental death moderates the association between out-of-home care trajectories and psychiatric disorders across adulthood. A major advantage of register data is the possibility of comparing outcomes between placed and non-placed individuals while adjusting for a comprehensive range of familial and individual confounding factors. By integrating developmental placement characteristics and life-course theoretical perspectives, the present study extends previous research beyond separate exposure models and investigates how cumulative and intersecting adversities may shape psychiatric risks across adulthood among individuals with experience of out-of-home care. The findings of the present study will contribute to a better understanding of how cumulative and intersecting adversities shape psychiatric risks in adulthood among individuals with experience of out-of-home care and may inform interventions targeting this highly vulnerable population.

## 2. Materials and Methods

### 2.1. Study Design

This study used nationwide Swedish register data linked through pseudonymized personal identification numbers (PINs), which are assigned to all residents from birth or the time of immigration to Sweden until death and allow reliable linkage across nationwide registers. The study population consisted of 10 cohorts born between 1972 and 1981 who resided in Sweden according to the Medical Birth Register administered by the National Board of Health and Welfare (NBHW) and who were alive in Sweden in 1985 according to the Population and Housing Census. Register information covered all individuals residing in Sweden from birth until death or until the end of follow-up on 31 December 2018 (ages 37–47), based on data from the Total Population Register administered by Statistics Sweden. Information on the relationships of the cohort members and their biological parents was obtained through linkage with the Multi-Generation Register administered by Statistics Sweden.

Access to register data required ethical approval and adherence to the data protection regulations of the respective register holders. No information enabling the identification of individual participants was available to the authors during any stage of data extraction or analysis. Ethical approval for the study was granted by the Swedish Ethical Review Authority (reference number: 2020-00250).

### 2.2. Population

The initial study population consisted of 992,361 individuals registered as residents of Sweden. Individuals with missing data due to incomplete information on parental sociodemographic information (range of missing data rates: 0.003–3.39%) were excluded. The final analytic sample thus comprised 944,138 individuals (459,712 women [48.70%] and 484,426 men [51.30%]), of which 23,106 individuals (2.45%; 11,074 women [2.41%] and 12,032 men [2.48%]) had experience of out-of-home care between the ages of 0 through 19.

### 2.3. Measures

Psychiatric disorders were the outcome and were identified using annual inpatient care records from the National Patient Register (administered by the NBHW) based on *International Classification of Diseases* (ICD; ICD-8, ICD-9, and ICD-10) codes. Binary indicators were created to reflect the presence or absence of the following diagnostic categories between ages 20 and 47: substance-use, schizophrenia/schizotypal, mood/affective, anxiety/stress-related and somatoform, behavioral/physiological syndromes, personality, and unspecified (see Supplemental Table S1). As the outcome relied on inpatient psychiatric records, the measures primarily captured more severe psychiatric conditions requiring specialized treatment and hospitalization. Consequently, milder, untreated, or exclusively outpatient-treated psychiatric disorders were not captured by this measure unless they resulted in inpatient admission. Additionally, since the primary aim of the present study was to investigate the association between a past out-of-home care experience and psychiatric disorders across adulthood, and because nationwide inpatient register coverage was incomplete before 1987, the analyses focused on psychiatric disorders occurring between ages 20 and 47, when nationwide coverage was considered comprehensive.

The primary exposure was out-of-home care, including compulsory or voluntary foster-family, kinship, or residential placements registered in the Child Welfare Intervention Register (administered by the NBHW). A binary variable was constructed to indicate whether an individual had experienced out-of-home care between birth and age 20 (0 = no; 1 = yes). Within the Swedish child welfare system, placements generally occur between birth and age 17 but may continue until age 21 in some compulsory cases or voluntary placements involving attendance in upper secondary school.

To capture heterogeneity in placement experiences, individuals with experience of out-of-home care were classified into distinct, non-overlapping groups reflecting different combinations of placement timing and duration (cf. Triseliotis [37]). This classification strategy has previously been applied in Swedish register-based cohort studies and has demonstrated strong discriminatory validity across a range of long-term outcomes [7,31,38,39,40]. The four placement groups were defined as follows [41]: 1 = early short-term: first placed before age 13 and spent less than one year in out-of-home care; 2 = early intermediate: first placed before age 13 and spent one to five years in out-of-home care; 3 = long-term: first placed before age 13 and spent more than five years in out-of-home care; 4 = teenage: first placed at age 13 or older, with any amount of time spent in out-of-home care.

For the early short-term, early intermediate, and long-term groups, placements typically reflect exposure to child maltreatment such as abuse or neglect. In contrast, teenage placements are more commonly associated with adolescents’ externalizing and behavioral difficulties, such as juvenile delinquency or substance misuse, and have historically represented the most common form of out-of-home placement in Sweden [7,42]. The long-term placement group is distinctive in that it includes children who spent a substantial proportion of their childhood in care, effectively growing up within the child welfare system [43]. In the Swedish context, prolonged placements of this kind often follow chronic family adversity and extended involvement with child welfare services rather than a single placement episode.

The main risk variable reflected the death of one or both parents (0 = no, 1 = yes) and was obtained from the Cause of Death Register administered by the NBHW. Parental death was assessed from ages 0 through 19 of a child’s life, corresponding to the developmental period during which out-of-home care could occur. Parental death and out-of-home care were both measured across childhood and adolescence; therefore, the timing of parental death may overlap with the timing and duration of placement. Thus, parental death may have occurred before, during, or after care had ended. The variable should be interpreted as an indicator of exposure to parental death across childhood and adolescence rather than as a temporally ordered event in relation to placement.

Children and adolescents placed in out-of-home care typically come from families with substantial socioeconomic and psychosocial burdens [44]. We adjusted for familial environmental confounding factors in the cohort members’ family of origin in our analyses. The selection of confounders followed prior Swedish register-based research [8,38,41]. Although it is challenging to identify pre-care familial characteristics from the data available, the selected measurements serve as adequate proxies for familial environmental conditions present in the cohort members’ family of origin.

Individual demographic information included sex (0 = male, 1 = female) and the birth year nominal variable, obtained from the Medical Birth Register administered by the NBHW.

Parental demographic characteristics and socioeconomic circumstances were obtained from the Longitudinal Integrated Database for Health and Labor Market Studies and the Population and Housing Census, both maintained by Statistics Sweden. Variables assessed for each parent included marital status (unmarried: 0 = no, 1 = yes), low socioeconomic status (0 = no, 1 = yes), educational attainment (0 = pre-secondary education, 1 = post-secondary education), and country of birth (0 = Sweden, 1 = Nordics, 2 = Europe, 4 = Other). The low socioeconomic status confounder was defined as having at least one record of receiving means-tested social assistance. This variable, together with parental education, was measured using the register data for 1990, when the cohort members were between 11 and 18 years old. Parental marital statuses were assessed once for each cohort member, between ages 4 and 8 years.

A psychiatric disorder confounder, in the form of a binary indicator (0 = no, 1 = yes), was determined for both parents using the Patient Register administered by the NBHW. A psychiatric disorder was considered present if there was a record of at least one inpatient admission with a primary or secondary psychiatric diagnosis, including substance-use disorders, according to the relevant ICD classification codes.

A binary indicator of criminality for each parent (0 = no, 1 = yes) was measured using the Conviction Register (administered by the Swedish Council for Crime Prevention) and defined as having at least one conviction resulting in a sentence of probation, imprisonment, or forensic psychiatric care. Information from the full observation period was used when assessing parental psychiatric disorders and parental criminality.

### 2.4. Statistical Analysis

All analyses were conducted in R version 4.4.1 [45]. Plots were created using the ggplot2 R package version 3.5.1. [46]. All analyses were stratified by sex assigned at birth. Sex-stratified analyses were conducted because previous research has consistently documented sex differences in psychiatric disorders [47] and out-of-home care, as well as related developmental outcomes [38]. Stratification was preferred over higher-order interaction models involving sex, as the main research question focused on whether parental death modified the association between out-of-home care and psychiatric disorders separately among women and men.

Descriptive information was presented as frequencies of psychiatric disorders and sociodemographic characteristics by out-of-home care and sex.

The survival probability for the outcome of any psychiatric disorder by placement characteristic group was explored using Kaplan–Meier plots separately for women and men [48]. Cox proportional hazard regression was employed to analyze the association between placement characteristic groups and psychiatric disorders, as well as between parental death and psychiatric disorders [49]. Individuals were entered on the day of their birth. The observational period for each individual began at age 20 and continued until either the date of death or the end of the follow-up period on 31 December 2018. Hazard ratios (HRs) with 95% confidence intervals (CIs) were calculated for two sets of models. The set consisted of a crude model, including placement characteristic group and parental death variables only. The second set comprised an adjusted model, additionally including all confounding factors in one model.

Statistical interaction analysis (effect measure modification) was performed to examine the moderation effect of parental death on the association between placement characteristic groups and psychiatric disorders (i.e., whether the association between one exposure and the outcome varied within strata of the other exposure). The main effect terms (placement characteristic group and parental death variables) as well as the interaction term (placement characteristic group × parental death) were included, after which predictive margins were computed. In addition, a comparison of model fit between the main effects model and the interaction model was conducted. If a likelihood ratio (LR) test produced a *p*-value < 0.05, the model with the interaction term was considered to fit the data better than a model without the interaction term. The proportional hazards assumption was evaluated using Schoenfeld residuals; there was no evidence of a violation of the proportional hazards assumption in any of the models. Adjusted predicted margins of psychiatric disorders by placement characteristic group and parental death status were plotted separately for women and men.

To test the robustness of the main findings, two sensitivity analyses were performed: First, to address the possibility that premature death may act as a competing event for psychiatric disorders, adjusted cause-specific Cox regression analyses were conducted, and adjusted Fine–Gray subdistribution hazard ratio models, including the competing risk of premature death and interaction effects in both models, were generated. The cause-specific hazard model estimates the effect of covariates on the rate at which events occur in individuals who are currently event-free [50]. Subdistribution hazard ratios obtained from the Fine–Gray model describe the relative effect of covariates on the subdistribution hazard function; the covariates of this model can also be interpreted as having an effect on the cumulative incidence function or on the probability of events occurring over time [50]. Second, adjusted Cox proportional hazard regression models including the interaction terms for all diagnostic categories were conducted separately in order to check whether the results changed depending on the diagnostic category.

## 3. Results

### 3.1. Descriptive Characteristics of the Population

Sex-stratified descriptive characteristics by out-of-home care status are reported in Table 1. The most common placement form was the teenage placement group for women (45.77%) and men (42.05%), followed by the early short-term, long-term, and early intermediate placement groups. The descriptive characteristics of different placement characteristic groups are presented in Supplemental Table S2 for women and Supplemental Table S3 for men.

The average age at first placement was 9.93 years (*SD* = 6.11, range = 0.04–20.95) for women and 9.75 years (*SD* = 6.12, range = 0.04–21.00) for men. The average total duration of care was 3.12 years (*SD* = 4.48, range = 0–21.01) for women and 3.31 years (*SD* = 4.46, range = 0–21.00) for men. In total, the average number of placements was 1.74 (*SD* = 1.34, range = 1–17) for women and 1.84 (*SD* = 1.34, range = 1–19) for men.

Placed women (23.23%) and men (26.18%) showed higher rates of any psychiatric disorder after age 20 compared to their non-placed counterparts (4.88% and 4.86%, respectively). Among out-of-home placed individuals, the highest rate was found for substance-use disorders among women (10.94%) and men (18.38%), followed by anxiety/stress-related and somatoform disorders (9.94% women, 7.11% men) and mood/affective disorders (7.67% women, 4.55% men).

Out-of-home placed women (5.26%) and men (5.28%) were more likely to experience the death of one or both parents from birth through 19 years of age compared to non-placed counterparts (1.93% and 1.95%, respectively). Moreover, placed individuals were more likely to experience premature death; their parents more often had low socioeconomic status and low educational attainment, were more often born outside the Nordic countries, were more often unmarried, and had higher rates of psychiatric disorders and criminality, compared to non-placed individuals.

### 3.2. Cox Proportional Hazard Regression Analysis

**Figure 1:**
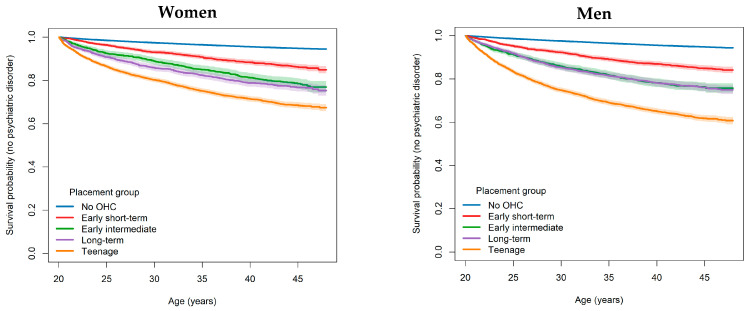
Kaplan–Meier curves showing the survival probability of remaining without psychiatric disorders across placement characteristic groups during the observation period, stratified by sex. OHC = out-of-home care.

Compared with non-placed individuals, those placed during adolescence showed the lowest survival probability over time, followed by those in long-term, early intermediate, and early short-term placements (Figure 1).

The sex-stratified proportional hazard regression analysis for the main effects of placement characteristic group and parental death is presented in Table 2. Among women, a higher risk of psychiatric disorders was found for all placement groups compared to non-placed women. Women placed as teenagers showed the highest risk (adjusted HR = 4.84, 95% CI: 4.58, 5.11) of psychiatric disorders, and early short-term placements had the smallest risk (adjusted HR = 1.55, 95% CI: 1.40, 1.72). A similar pattern was observed for men: placed men showed a higher risk of psychiatric disorders compared to non-placed men, with the highest risk for teenage placements (adjusted HR = 5.66, 95% CI: 5.38, 5.95) and the smallest risk for early short-term placements (adjusted HR = 1.56, 95% CI: 1.42, 1.72). Women (adjusted HR = 1.17, 95% CI: 1.09, 1.26) and men (HR = 1.19, 95% CI: 1.11, 1.28) had a higher risk of psychiatric disorders if they experienced the death of one or both parents from birth through age 19 compared to their counterparts without parental death.

The sex-stratified proportional hazard regression analysis for the main and interaction effects of placement characteristic groups and parental death is reported in Table 3. The results of the main effects of both placement characteristic groups and parental death on psychiatric disorders remained consistent with the results from the models with main effects only. Out-of-home placed individuals showed higher risk of psychiatric disorders compared to non-placed individuals, with the highest risk for teenage placements (women: adjusted HR = 4.98, 95% CI: 4.71, 5.27; men: adjusted HR = 5.81, 95% CI: 5.52, 6.12) and lowest risk for early short-term placements (women: adjusted HR = 1.59, 95% CI: 1.43, 1.77; men: adjusted HR = 1.66, 95% CI: 1.46, 1.78). Compared to individuals without an experience of parental death, women (adjusted HR = 1.31, 95% CI: 1.21, 1.42) and men (HR = 1.33, 95% CI: 1.23, 1.44) had a higher risk of psychiatric disorders if they experienced the death of one or both parents from birth through age 19.

Significant interaction effects between some placement characteristic groups and parental death were found. Among women, the association between parental death and psychiatric disorder was lower for teenage (adjusted HR = 0.53, 95% CI: 0.41, 0.70), long-term (adjusted HR = 0.61, 95% CI: 0.38, 0.96), and early intermediate (adjusted HR = 0.63, 95% CI: 0.40, 0.99) placement groups compared to non-placed women. Among men, the relationship between parental death and psychiatric disorder was lower for the early intermediate (adjusted HR = 0.53, 95% CI: 0.33, 0.85), teenage (adjusted HR = 0.59, 95% CI: 0.47, 0.75), and early short-term (adjusted HR = 0.59, 95% CI: 0.38, 0.92) placement groups compared to non-placed men. The adjusted models including the interaction term had a significantly improved model fit compared to the adjusted models without the interaction term (LR test women: χ^2^(4) = 33.26, *p* < 0.001; LR test men: χ^2^(4) = 31.09, *p* < 0.001).

**Figure 2:**
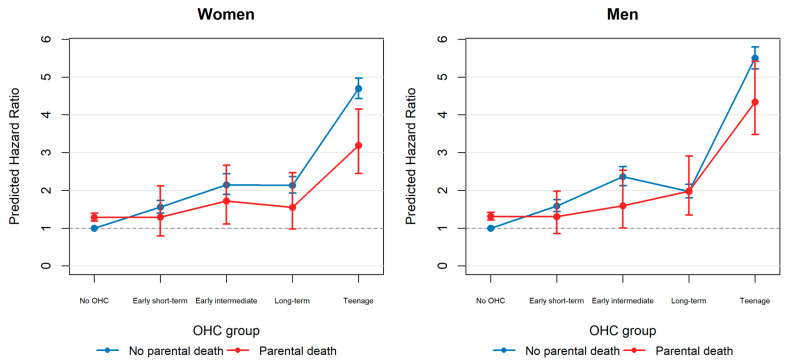
Adjusted predicted margins plot of psychiatric disorders by placement characteristic group and parental death status with 95% confidence intervals, stratified by sex. The adjusted model controlled for parental birth country, parental psychiatric disorder, parental criminality, parental marital status, parental low socioeconomic status, and parental low educational achievement. OHC = out-of-home care.

The results for the predicted hazards derived from the Cox regression models are presented in Figure 2. Across both sexes, placed individuals showed substantially higher hazards of psychiatric disorders compared with those without out-of-home care placement, regardless of parental death status. Within placement groups, differences between individuals with and without parental death varied in magnitude, and, in some placement groups, the predicted hazards for individuals who experienced parental death were similar to or lower than those without parental death. Parental death was associated with increased hazards in the reference group of those without out-of-home care placement, whereas its relative contribution was attenuated among placed individuals. This pattern is consistent with the interaction effects observed in the Cox regression models, indicating that the relative impact of parental death differs across placement characteristic groups.

Sensitivity analyses accounting for the competing risk of premature death using cause-specific Cox regression and Fine–Gray models (Supplemental Tables S4 and S5) showed findings comparable to those of the main Cox proportional hazard regression analyses including the interaction term. Additionally, the overall pattern observed for any psychiatric disorder was largely replicated across different diagnostic categories, with consistently higher risks observed for teenage placements (Supplemental Tables S6–S11). The effect sizes were particularly pronounced for substance-use and personality disorders, whereas effects were weaker and less precise for behavioral/physiological syndromes. The interaction effects between placement characteristic groups and parental death were less consistent across diagnostic categories and were imprecisely estimated due to low event counts in specific strata. Overall, the sensitivity analyses supported the robustness of the main results.

## 4. Discussion

This Swedish cohort study was the first to use register data of almost 1,000,000 individuals born between 1972 and 1981, of whom around 2.5% had experience of out-of-home care, to examine the association between out-of-home care and parental death during childhood and adolescence and psychiatric disorders in adulthood.

The findings showed that individuals with experience of out-of-home care had approximately five times higher rates of psychiatric disorders compared to non-placed individuals. The prevalence rates reported in our study fall within the range of the rates reported in a previous meta-analysis showing that almost one third of adults with a history of out-of-home care had a mental disorder, with rates higher compared with those for non-placed individuals [16]. Additionally, the pattern of rates for the different diagnostic categories found in our study (i.e., highest rates for substance-use disorders, followed by anxiety/stress-related and somatoform disorders and mood/affective disorders) also aligns with the pattern reported in previous studies in out-of-home placed populations [20,51]. Together, these findings support previous research, suggesting that children and adolescents in out-of-home care are a mentally vulnerable group susceptible to the accumulation of familial and psychosocial adversities.

Individuals with experience of out-of-home care had almost threefold higher rates of experiencing the death of one or both parents from birth through age 19 compared to non-placed individuals. This pattern aligns with previous evidence showing that early parental death is overrepresented among individuals with experience of out-of-home care compared to the general population [32]. Early parental death further increased the risk of psychiatric disorders compared to individuals with both parents alive in childhood and adolescence, which is in line with previous evidence showing that early parental separation, particularly due to death, is a risk factor for poor mental health [24,25,26]. From a developmental perspective, early parental loss and separation can thus lead to long-term mental health consequences. Moreover, experiences of parental loss during sensitive developmental periods may influence attachment formation, emotional regulation, and perceptions of safety differently across the life course. The markedly elevated prevalence of parental death among individuals with experience of out-of-home care is therefore consistent with an accumulation of severe and intersecting adversities in this already vulnerable population.

The main findings of the present study showed that the relative association between parental death and psychiatric disorders was attenuated among individuals with experience of out-of-home care compared to non-placed individuals. Although psychiatric risks remained substantially elevated among placed individuals irrespective of parental death status, the additional relative contribution of parental death appeared smaller within placement groups. The magnitude of this attenuation varied depending on placement timing and duration, with the lowest relative risks observed among teenage placements. Adolescents in Sweden are typically placed due to externalizing difficulties and disruptive behavior (e.g., delinquency or substance misuse) [7], which may reflect already high levels of psychosocial burden and accumulated adversity prior to placement. While teenage placements may reflect developmental pathways characterized by behavioral dysregulation and psychosocial instability, early childhood placements may more often reflect chronic family dysfunction and prolonged exposure to neglect or maltreatment. From a theoretical perspective, the findings may therefore be more consistent with the disadvantage-saturation perspective than with a cumulative disadvantage perspective, which assumes additive psychiatric risks associated with exposure to both parental death and out-of-home care. However, these findings should not be interpreted as definitive confirmation of the disadvantage-saturation theory, given the observational nature of the study and the possibility of alternative explanations underlying the attenuated interaction effects.

The attenuated relative effect of parental death among individuals with experience of out-of-home care may also reflect ceiling effects associated with already elevated baseline psychiatric risks, statistical compression at high levels of disadvantage, or differences in healthcare access and detection patterns in out-of-home placed populations. In addition, the parent–child relationship in out-of-home care settings may be emotionally complex and characterized by attachment disruptions, conflicting loyalties, or ambivalent family relationships, potentially influencing how parental loss is experienced [4,21,35]. The developmental timing of adversity exposure may be important when examining psychiatric vulnerability among individuals with experience of out-of-home care. As parental death was measured between ages 0 and 19 years, it may represent both a pathway into care and an event during or after care. The observed attenuation therefore likely reflects the cumulative co-occurrence of adversities across different developmental stages rather than the isolated effect of parental death at a single developmental time point. Future research should therefore investigate whether parental death, occurring during different developmental periods (e.g., early childhood, middle childhood, or adolescence), differentially influences long-term psychiatric vulnerability and developmental adjustment among individuals with experience of out-of-home care.

The findings of the present study are relevant for child welfare services, mental health professionals, and public health practitioners working with out-of-home placed individuals. The findings highlight the substantial psychiatric burden among individuals with experience of out-of-home care, irrespective of parental death status. The results should therefore not be interpreted as suggesting that parental death is clinically unimportant to individuals in care. Rather, they indicate that psychiatric vulnerability in this population is shaped by multiple intersecting adversities requiring comprehensive and integrated intervention approaches. Furthermore, the findings suggest that interventions targeting isolated adverse experiences alone may be insufficient for highly disadvantaged populations, such as out-of-home placed individuals, characterized by psychosocial stressors. Mental health services may need to adopt more integrated, long-term, and trauma-informed approaches that simultaneously address cumulative adversity and broader psychosocial functioning across developmental stages. Moreover, early placements for children who experienced early adversities and parental loss might be a crucial prevention approach to mitigate later psychiatric risk. Particular attention may be warranted for adolescents entering care, who showed the highest psychiatric risks in adulthood. Additionally, systematic mental health screening, grief-informed support, attachment-focused approaches, and continuity of psychiatric care during the transition from adolescence to adulthood may be particularly important for reducing long-term psychiatric vulnerability. These findings may also support the development of coordinated cross-sector interventions involving child welfare services and mental healthcare systems in order to improve continuity of support for young people with complex and cumulative adversity exposure. Together, timely and integrated interventions addressing cumulative adversities and psychopathology may thus help reduce the long-term burden of chronic psychiatric disorders and associated public health consequences in this highly vulnerable population.

### Strengths and Limitations

This Swedish population-based cohort study has a number of strengths, including a large representative sample of almost 1,000,000 individuals that enables the investigation of smaller population groups and rare events such as diagnostic categories. The use of register-based data minimized attrition and bias, enhancing the reliability of the findings. Additionally, the prospective psychiatric records enabled us to follow individuals over a long period of follow-up (i.e., ages 20 to 47), providing a long-term perspective on psychiatric risks in adulthood and improving the validity of the data. The rich nature of the register data also allowed us to control for a comprehensive set of parental confounding factors, known to be crucial markers of selection into out-of-home care.

The findings of the present study need to be interpreted in the light of some limitations. Register-based data do not fully capture the complex processes underlying the observed associations between out-of-home care, parental death, and psychiatric disorders. Although the analyses controlled for several important parental and familial confounders, residual confounding by unmeasured factors remains possible. For example, information on attachment representations, temperament, quality of care, and family relationship quality was not available in the registers. Moreover, future studies may benefit from applying multilevel or family-based modeling approaches to further account for clustering within families or contextual variation across care settings. Additionally, the registers only recorded the type of first placement, limiting the possibility of distinguishing between foster-family and residential placements across the full placement history. In the Swedish child welfare system, however, foster-family placements are the most common form of out-of-home care, especially for children placed in middle childhood, while residential care is less frequent and largely applies to adolescents. Thus, the distinction may be partly reflected in the adolescent placement variable. Access to such data would have provided a deeper understanding of how different placement conditions and experiences influence psychiatric trajectories across the life course. Finally, due to the observational nature of this study design, the associations of out-of-home care and parental death with psychiatric risks do not imply causality.

Psychiatric disorders were identified using inpatient care records and therefore primarily captured more severe psychiatric conditions requiring specialized inpatient care. Mild, untreated, or exclusively outpatient-treated psychiatric disorders were thus likely underestimated. Accordingly, these findings should be interpreted as reflecting risks of more severe forms of psychiatric morbidity rather than the full spectrum of mental health issues in the population. In addition, differences in healthcare access or contact with psychiatric services across population groups may have contributed to differential detection and classifications of psychiatric disorders. However, the disorder-specific sensitivity analyses largely replicated the overall pattern of findings across diagnostic categories. Additionally, individuals with experience of out-of-home care may have had greater contact with healthcare services, potentially increasing the likelihood that severe psychiatric conditions were identified and recorded in inpatient registers compared to individuals without experience of out-of-home care.

The temporal ordering between parental death and out-of-home care placement also could not be determined precisely because parental death was measured across the entire age period from 0 through 19 years, which overlaps with the period during which out-of-home care could occur. Parental death may therefore have occurred before, during, or after care had ended. This limits the possibility of determining whether parental death functioned as a pathway into care, an event experienced while in care, or an adversity occurring after care. Consequently, the findings should be interpreted as reflecting the cumulative co-occurrence of adversities across childhood and adolescence rather than a specific sequence between parental death and out-of-home care. Future studies should therefore examine the impact of early parental death on the development across the life course and different developmental stages.

Socioeconomic and systemic factors (e.g., socioeconomic status, marital status, quality of life) in adulthood were not considered and may also contribute to psychiatric outcomes later in life. Examining adult adjustment among out-of-home placed individuals who had experienced early parental death was beyond the scope of this study and should be addressed in future research.

Finally, the generalizability of the findings of this study to populations outside of Sweden and to more recent cohorts may be limited due to the Swedish context, including its child protection services and healthcare system, and given that the study population includes individuals born between 1972 and 1981. However, no changes in the child welfare system or register data collection methods occurred during the observation period for this study, which strengthens the reliability of the study findings.

## 5. Conclusions

The findings of the present study showed that parental death attenuated the relative association between out-of-home care and psychiatric disorders in adulthood. Individuals with experience of out-of-home care are marked by substantially elevated psychiatric risks irrespective of parental death status, reflecting the accumulation of multiple adversities across childhood and adolescence. Although the findings may be consistent with the disadvantage-saturation perspective, they should not be interpreted as confirming this mechanism. Alternative explanations, including ceiling effects, differences in healthcare access or detection, and the parent–child relationship in out-of-home care settings, should also be considered.

From a public health perspective, the findings emphasize the substantial psychiatric vulnerability among individuals with experience of out-of-home care and highlight the importance of integrated and developmentally sensitive interventions addressing cumulative adversity rather than isolated adverse experiences alone. Trauma-informed mental health services, grief-informed support, attachment-focused interventions, and continuity of care during developmental transitions may be particularly important for reducing the long-term burden of psychiatric disorders among children and adolescents in care.

## Figures and Tables

**Table 1:** Sample characteristics by out-of-home care experience, stratified by sex (*N*/%).

Variables	Women (*n* = 459,712/48.70)		Men (*n* = 484,426/51.30)	
	Out-of-Home Placed (*n* = 11,074/2.41)	Home (*n* = 448,638/97.59)	Out-of-Home Placed (*n* = 12,032/2.48)	Home (*n* = 472,394/97.52)
Placement characteristic group				
No placement	N/A	448,638 (100.00)	N/A	472,394 (100)
Early short-term	2775 (25.06)	N/A	2999 (24.93)	N/A
Early intermediate	1372 (12.39)	N/A	1744 (14.49)	N/A
Long-term	2060 (18.60)	N/A	2416 (20.08)	N/A
Teenage placement	5069 (45.77)	N/A	5059 (42.05)	N/A
Parental death (0–19 years)	583 (5.26)	8646 (1.93)	635 (5.28)	9216 (1.95)
Any psychiatric disorder (F10–69)	2572 (23.23)	21,888 (4.88)	3150 (26.18)	22,980 (4.86)
Substance-use disorders (F10–F19)	1212 (10.94)	6226 (1.39)	2212 (18.38)	11,132 (2.36)
Schizophrenia/schizotypal disorders (F20–F29)	291 (2.63)	2436 (0.54)	507 (4.21)	3468 (0.73)
Mood/affective disorders (F30–F39)	849 (7.67)	8442 (1.88)	548 (4.55)	6328 (1.34)
Anxiety/stress-related and somatoform disorders (F40–F48)	1101 (9.94)	8772 (1.96)	855 (7.11)	6607 (1.40)
Behavioral/physiological syndromes (F50–F59)	138 (1.25)	1789 (0.4)	42 (0.35)	382 (0.08)
Personality disorders (F60–F69)	497 (4.49)	2147 (0.48)	363 (3.02)	1171 (0.25)
Premature death	299 (2.70)	3490 (0.78)	882 (7.33)	7504 (1.59)
Paternal low socioeconomic status	3265 (29.48)	20,636 (4.60)	3691 (30.68)	21,777 (4.61)
Maternal low socioeconomic status	5034 (45.46)	23,875 (5.32)	5426 (45.10)	25,218 (5.34)
Paternal marital status (unmarried)	6432 (58.08)	100,970 (22.51)	6951 (57.77)	105,855 (22.41)
Maternal marital status (unmarried)	6512 (58.80)	102,190 (22.78)	7092 (58.94)	107,217 (22.70)
Paternal birth country				
Sweden	8838 (79.81)	400,367 (89.24)	9383 (77.98)	421,776 (89.28)
Nordics	1010 (9.12)	18,998 (4.23)	1111 (9.23)	19,934 (4.22)
Europe	763 (6.89)	20,906 (4.66)	943 (7.84)	21,852 (4.63)
Other	463 (4.18)	8367 (1.86)	595 (4.95)	8832 (1.87)
Maternal birth country				
Sweden	9035 (81.59)	401,898 (89.58)	9687 (80.51)	422,959 (89.54)
Nordics	1167 (10.54)	23,641 (5.27)	1229 (10.21)	25,051 (5.30)
Europe	560 (5.06)	16,449 (3.67)	731 (6.08)	17,401 (3.68)
Other	312 (2.82)	6650 (1.48)	385 (3.20)	6983 (1.48)
Paternal low educational achievement (pre-secondary education)	5119 (46.23)	144,601 (32.23)	5581 (46.38)	151,962 (32.17)
Maternal low educational achievement (pre-secondary education)	5183 (46.8)	119,845 (26.71)	5565 (46.25)	125,758 (26.62)
Paternal psychiatric disorder	4161 (37.57)	52,207 (11.64)	4624 (38.43)	54,021 (11.44)
Paternal criminality	459 (4.14)	3287 (0.73)	453 (3.76)	3358 (0.71)
Maternal psychiatric disorder	4888 (44.14)	37,778 (8.42)	4990 (41.47)	40,125 (8.49)
Maternal criminality	161 (1.45)	398 (0.09)	182 (1.51)	412 (0.09)

Note. N/A = not applicable. Early short-term = placed for the first time < age 13 and with <1 year in total in out-of-home care (OHC) < age 18. Early intermediate = placed for the first time < age 13 and with 1–5 years in total in OHC < age 18. Long-term = placed for the first time < age 13 and with >5 years in total in OHC < age 18. Teenage placement = placed for the first time ≥ age 13, regardless of total time in OHC < age 18.

**Table 2:** Main effects of the Cox proportional hazard regression analysis of placement characteristic group, parental death, and psychiatric disorder, stratified by sex.

Variables	Women (*n* = 459,712/48.70)		Men (*n* = 484,426/51.30)	
	HR (95% CI)	HR (95% CI)	HR (95% CI)	
	Crude	Adjusted	Crude	Adjusted
Placement characteristic group				
Early short-term	2.79 (2.52, 3.09)	1.55 (1.40, 1.72)	3.09 (2.81, 3.40)	1.56 (1.42, 1.72)
Early intermediate	4.53 (4.03, 5.09)	2.10 (1.86, 2.37)	5.37 (4.87, 5.93)	2.33 (2.10, 2.58)
Long-term	5.15 (4.70, 5.65)	2.13 (1.93, 2.35)	5.33 (4.90, 5.80)	1.97 (1.80, 2.16)
Teenage placement	7.80 (7.41, 8.21)	4.84 (4.58, 5.11)	10.08 (9.62, 10.57)	5.66 (5.38, 5.95)
Parental death	1.36 (1.26, 1.46)	1.17 (1.09, 1.26)	1.43 (1.33, 1.53)	1.19 (1.11, 1.28)

Note. *HR* = hazard ratio, *CI* = confidence interval. Reference for placement characteristic group: o out-of-home care. Reference category parental death: no parental death. The crude model included the main effects of the placement characteristic group and parental death variables. The adjusted model controlled for parental birth country, parental psychiatric disorder, parental criminality, parental marital status, parental low socioeconomic status, and parental low educational achievement. Early short-term = placed for the first time < age 13 and with <1 year in total in out-of-home care (OHC) < age 18. Early intermediate = placed for the first time < age 13 and with 1–5 years in total in OHC < age 18. Long-term = placed for the first time < age 13 and with >5 years in total in OHC < age 18. Teenage placement = placed for the first time ≥ age 13, regardless of total time in OHC < age 18.

**Table 3:** Main and interaction effects of the Cox proportional hazard regression analysis of placement characteristic groups, parental death, and psychiatric disorders, stratified by sex.

Variables	Women (*n* = 459,712/48.70)	Men (*n* = 484,426/51.30)
	Adjusted HR (95% CI)	Adjusted HR (95% CI)
Placement characteristic group		
Early short-term	1.59 (1.43, 1.77)	1.61 (1.46, 1.78)
Early intermediate	2.18 (1.92, 2.47)	2.42 (2.18, 2.68)
Long-term	2.18 (1.97, 2.41)	2.01 (1.83, 2.20)
Teenage placement	4.98 (4.71, 5.27)	5.81 (5.52, 6.12)
Parental death	1.31 (1.21, 1.42)	1.33 (1.23, 1.44)
Interaction between placement characteristic group and parental death		
Early short-term × parental death	0.63 (0.38, 1.04)	0.59 (0.38, 0.92)
Early intermediate × parental death	0.63 (0.40, 0.99)	0.53 (0.33, 0.85)
Long-term × parental death	0.61 (0.38, 0.96)	0.69 (0.46, 1.05)
Teenage placement × parental death	0.53 (0.41, 0.70)	0.59 (0.47, 0.75)

Note. *HR* = hazard ratio, *CI* = confidence interval. Reference for placement characteristic group: no out-of-home care. Reference category parental death: no parental death. The adjusted model controlled for parental birth country, parental psychiatric disorder, parental criminality, parental marital status, parental low socioeconomic status, and parental low educational achievement. Early short-term = placed for the first time < age 13 and with <1 year in total in out-of-home care (OHC) < age 18. Early intermediate = placed for the first time < age 13 and with 1–5 years in total in OHC < age 18. Long-term = placed for the first time < age 13 and with >5 years in total in OHC < age 18. Teenage placement = placed for the first time ≥ age 13, regardless of total time in OHC < age 18.

## Data Availability

The data cannot be made publicly available due to the sensitive nature of the data and the Swedish data protection legislation. Researchers may apply for access through Statistics Sweden, the Swedish National Council for Crime Prevention, and the National Board of Health and Welfare.

## Supplementary Materials

The following supporting information can be downloaded at https://www.mdpi.com/article/10.3390/ijerph23060732/s1. Table S1: Included and excluded F-Codes from the International Classification of Diseases (ICD); Table S2: Sample characteristics by placement characteristic group, women (*n* = 459,712) (*N*/%); Table S3: Sample characteristics by placement characteristic group, men (*n* = 484,426) (*N*/%); Table S4: Main and interaction effects of the cause-specific Cox regression analysis of placement characteristic group, parental death, and psychiatric disorder, stratified by sex; Table S5: Main and interaction effects of the Fine–Gray subdistribution hazard ratio (SHR) model of placement characteristic group, parental death, and psychiatric disorder, stratified by sex; Table S6: Main and interaction effects of the Cox proportional hazard regression analysis of placement characteristic group, parental death, and substance-use disorders, stratified by sex; Table S7: Main and interaction effects of the Cox proportional hazard regression analysis of placement characteristic group, parental death, and schizophrenia/schizotypal disorders, stratified by sex; Table S8: Main and interaction effects of the Cox proportional hazard regression analysis of placement characteristic group, parental death, and mood/affective disorders, stratified by sex; Table S9: Main and interaction effects of the Cox proportional hazard regression analysis of placement characteristic group, parental death, and anxiety/stress-related and somatoform disorders, stratified by sex; Table S10: Main and interaction effects of the Cox proportional hazard regression analysis of placement characteristic group, parental death, and behavioral/physiological syndromes, stratified by sex; Table S11: Main and interaction effects of the Cox proportional hazard regression analysis of placement characteristic group, parental death, and personality disorders, stratified by sex.

## Abbreviations

The following abbreviations are used in this manuscript:

PIN	Personal Identification Numbers
NBHW	National Board of Health and Welfare
ICD	International Classification of Diseases
HRs	Hazard Ratios
CIs	Confidence Intervals
LR	Likelihood Ratio
