# Supplementary material for: Parental Death and Psychiatric Disorders Among Individuals with and Without Experience of Out-of-Home Care: A Swedish Nationwide Cohort Study
Source: Int J Environ Res Public Health. 2026 May 30;23(6):732. doi: 10.3390/ijerph23060732 (PMC13299293; doi:10.3390/ijerph23060732)
Supplement: Supplementary file 1 [file ijerph-23-00732-s001.zip › ijerph-4298494-supplementary.pdf]

Supplementary Material

Table S1.
Included and excluded F-Codes from the International Classification of Diseases (ICD).

**Disorder category**	***ICD-10***	***ICD-8/9***
**Included**		
Substance-use	F10-F19	291, 292, 303-305
Schizophrenia/schizotypal	F20-F29	295-298
Mood/affective	F30-F39	296, 300.4, 311
Anxiety/stress-related and somatoform	F40-F48	300 (excluding 300.4), 308-309
Behavioral/physiological	F50-F59	302, 306-307
Personality	F60-F69	301
Unspecified	F99	n/a
**Excluded**		
Organic	F00-F09	290, 293, 294, 310
Intellectual/developmental/childhood-onset	F70-F98	299, 312-315, 317-319

Table S2.
Sample characteristics by placement characteristic group, women (*n* = 459,712) (*N*/%).

	No out-of-home care (*n* = 448,638)	Early short-term (*n* = 2,775)	Early intermediate (*n* = 1,372)	Long-term (*n* = 2,031)	Teenage placement (*n* = 4,929)
Parental death (0–19 years)	8,646 (1.93)	122 (4.42)	111 (8.19)	100 (4.92)	250 (5.07)
Any psychiatric disorder (F10-69)	21,888 (4.88)	364 (13.2)	277 (20.43)	452 (22.26)	1,479 (30.01)
Premature death	3,490 (0.78)	49 (1.78)	34 (2.51)	62 (3.05)	154 (3.12)
Paternal low socioeconomic status	20,636 (4.60)	719 (26.07)	480 (35.40)	813 (40.03)	1,253 (25.42)
Maternal low socioeconomic status	23,875 (5.32)	1,105 (40.07)	773 (57.01)	1,257 (61.89)	1,899 (38.53)
Paternal marital status (unmarried)	100,970 (22.51)	1,584 (57.43)	921 (67.92)	1,479 (72.82)	2,448 (49.67)
Maternal marital status (unmarried)	102,190 (22.78)	1,614 (58.52)	918 (67.70)	1,488 (73.26)	2,492 (50.56)
Paternal birth country					
Sweden	400,367 (89.24)	2,065 (74.87)	1,088 (80.24)	1,721 (84.74)	3,964 (80.42)
Nordics	18,998 (4.23)	274 (9.93)	133 (9.81)	194 (9.55)	409 (8.30)
Europe	20,906 (4.66)	273 (9.90)	80 (5.90)	73 (3.59)	337 (6.84)
Other	8,367 (1.86)	146 (5.29)	55 (4.06)	43 (2.12)	219 (4.44)
Maternal birth country					
Sweden	401,898 (89.58)	2,112 (76.58)	1,123 (82.82)	1,770 (87.15)	4,030 (81.76)
Nordics	23,641 (5.27)	320 (11.6)	156 (11.50)	189 (9.31)	502 (10.18)
Europe	16,449 (3.67)	221 (8.01)	48 (3.54)	47 (2.31)	244 (4.95)
Other	6,650 (1.48)	105 (3.81)	29 (2.14)	25 (1.23)	153 (3.10)
Paternal low educational achievement (pre-secondary education)	144,601 (32.23)	1,278 (46.34)	642 (47.35)	1,111 (54.70)	2,088 (42.36)
Maternal low educational achievement (pre-secondary education)	119,845 (26.71)	1,236 (44.82)	677 (49.93)	1,237 (60.91)	2,033 (41.25)
Paternal psychiatric disorder	52,207 (11.64)	969 (35.13)	604 (44.54)	1,076 (52.98)	1,512 (30.68)
Paternal criminality	3,287 (0.73)	97 (3.52)	66 (4.87)	135 (6.65)	161 (3.27)
Maternal psychiatric disorder	37,778 (8.42)	1,205 (43.69)	780 (57.52)	1,347 (66.32)	1,556 (31.57)
Maternal criminality	398 (0.09)	33 (1.20)	24 (1.77)	60 (2.95)	44 (0.89)

Note. Early short-term = placed for the first time < age 13 and with < 1 year in total in out-of-home care (OHC) < age 18. Early intermediate = placed for the first time < age 13 and with 1–5 years in total in OHC < age 18. Long-term = placed for the first time < age 13 and with > 5 years in total in OHC < age 18. Teenage placement = placed for the first time ≥ age 13, regardless of total time in OHC < age 18.

Table S3.
Sample characteristics by placement characteristic group, men (*n* = 484,426) (*N*/%).

	No out-of-home care (*n* = 472,394)	Early short-term (*n* = 2,982)	Early intermediate (*n* = 1,725)	Long-term (*n* = 2,401)	Teenage placement (*n* = 4,924)
Parental death (0–19 years)	9,216 (1.95)	171 (5.73)	104 (6.03)	110 (4.58)	250 (5.08)
Any psychiatric disorder (F10-69)	22,980 (4.86)	429 (14.39)	391 (22.67)	554 (23.07)	1,776 (36.07)
Premature death	7,504 (1.59)	121 (4.06)	117 (6.78)	134 (5.58)	510 (10.36)
Paternal low socioeconomic status	21,777 (4.61)	750 (25.15)	573 (33.22)	965 (40.19)	1,403 (28.49
Maternal low socioeconomic status	25,218 (5.34)	1,164 (39.03)	915 (53.04)	1,418 (59.06)	1,929 (39.18)
Paternal marital status (unmarried)	105,855 (22.41)	1,717 (57.58)	1,142 (66.20)	1,691 (70.43)	2,401 (48.76)
Maternal marital status (unmarried)	107,217 (22.70)	1,784 (59.83)	1,148 (66.55)	1,713 (71.35)	2,447 (49.70)
Paternal birth country					
Sweden	421,776 ((89.28)	2,247 (75.35)	1,349 (78.2)	2,018 (84.05)	3,769 (76.54)
Nordics	19,934 (4.22)	279 (9.36)	160 (9.28)	234 (9.75)	438 (8.90)
Europe	21,852 (4.63)	302 (10.13	124 (7.19)	91 (3.79)	426 (8.65)
Other	8,832 (1.87)	154 (5.16)	92 (5.33)	58 (2.42)	291 (5.91
Maternal birth country					
Sweden	422,959 (89.54)	2,314 (77.60)	1,402 (81.28)	2,074 (86.38)	3,897 (79.14)
Nordics	25,051 (5.30)	310 (10.40)	183 (10.61)	245 (10.20)	491 (9.97)
Europe	17,401 (3.68)	244 (8.18)	88 (5.10)	52 (2.17)	347 (7.05)
Other	6,983 (1.48)	114 (3.82)	52 (3.01)	30 (1.25)	189 (3.84)
Paternal low educational achievement (pre-secondary education)	151,962 (32.17)	1,335 (44.77)	778 (45.1)	1,284 (53.48)	2,184 (44.35)
Maternal low educational achievement (pre-secondary education)	125,758 (26.62)	1,684 (43.53)	919 (46.72)	1,033 (56.98)	2,831 (42.51)
Paternal psychiatric disorder	54,021 (11.44)	1,043 (34.98)	713 (41.33)	1,248 (51.98)	1,620 (32.90)

Paternal criminality	3,358 (0.71)	100 (3.35)	73 (4.23)	123 (5.12)	157 (3.19)
Maternal psychiatric disorder	40,125 (8.49)	1,237 (41.48)	870 (50.43)	1,506 (62.72)	1,377 (27.97)
Maternal criminality	412 (0.09)	25 (0.84)	27 (1.57)	81 (3.37)	49 (1.00)

Note. Early short-term = placed for the first time < age 13 and with < 1 year in total in out-of-home care (OHC) < age 18. Early intermediate = placed for the first time < age 13 and with 1–5 years in total in OHC < age 18. Long-term = placed for the first time < age 13 and with > 5 years in total in OHC < age 18. Teenage placement = placed for the first time ≥ age 13, regardless of total time in OHC < age 18.

**Table S4.**<br>Main and interaction effects of the cause-specific Cox regression analysis of placement characteristic group, parental death, and psychiatric disorder, stratified by sex.		
	Women (*n* = 459,712/48.70)	Men (*n* = 484,426/51.30)
Variables	Adjusted HR (95% CI)	Adjusted HR (95% CI)
Placement characteristic group		
Early short-term	1.56 (1.40, 1.73)	1.59 (1.44, 1.76)
Early intermediate	2.15 (1.89, 2.44)	2.36 (2.13, 2.63)
Long-term	2.13 (1.93, 2.36)	1.97 (1.80, 2.16)
Teenage placement	4.69 (4.43, 4.97)	5.50 (5.21, 5.80)
Parental death	1.29 (1.18, 1.39)	1.31 (1.22, 1.42)
Interaction between placement characteristic group and parental death		
Early short-term × parental death	0.65 (0.39, 1.08)	0.63 (0.40, 0.97)
Early intermediate × parental death	0.62 (0.39, 0.99)	0.52 (0.32, 0.83)
Long-term × parental death	0.57 (0.35, 0.91)	0.76 (0.51, 1.14)
Teenage placement × parental death	0.53 (0.40, 0.70)	0.60 (0.47, 0.76)
Note. HR = hazard ratio, *CI* = confidence interval. The reference category included those participants without any record of psychiatric disorder (women: *n* = 435,252, men: *n* = 458,296). Reference for placement characteristic groups: no out-of-home care. Reference category parental death: no parental death. The adjusted model controlled for parental psychiatric disorder, parental criminality, parental marital status, parental low socioeconomic status, and parental low educational achievement.<br>Early short-term = placed for the first time < age 13 and with < 1 year in total in out-of-home care (OHC) < age 18. Early intermediate = placed for the first time < age 13 and with 1–5 years in total in OHC < age 18. Long-term = placed for the first time < age 13 and with > 5 years in total in OHC < age 18. Teenage placement = placed for the first time ≥ age 13, regardless of total time in OHC < age 18.		

**Table S5.**<br>Main and interaction effects of the Fine–Gray subdistribution hazard ratio (SHR) model of placement characteristic group, parental death, and psychiatric disorder, stratified by sex.		
	Women (*n* = 459,712/48.70)	Men (*n* = 484,426/51.30)
Variables	Adjusted SHR (95% CI)	Adjusted SHR (95% CI)
Placement characteristic group		
Early short-term	1.55 (1.39, 1.73)	1.58 (1.42, 1.75)
Early intermediate	2.15 (1.89, 2.44)	2.34 (2.10, 2.61)
Long-term	2.12 (1.91, 2.36	1.96 (1.78, 2.16)
Teenage placement	4.67 (4.39, 4.97)	5.37 (5.07, 5.69)
Parental death	1.28 (1.18, 1.39)	1.31 (1.21, 1.41)
Interaction between placement characteristic group and parental death		
Early short-term × parental death	0.65 (0.39, 1.07)	0.62 (0.39, 0.96)
Early intermediate × parental death	0.63 (0.39, 1.00)	0.51 (0.32, 0.83)
Long-term × parental death	0.57 (0.35, 0.92)	0.77 (0.51, 1.15)
Teenage placement × parental death	0.53 (0.40, 0.71)	0.60 (0.46, 0.78)
Note. HR = hazard ratio, *CI* = confidence interval. The reference category included those participants without any record of psychiatric disorder (women: *n* = 435,252, men: *n* = 458,296). Reference for placement characteristic groups: no out-of-home care. Reference category parental death: no parental death. The adjusted		

model controlled for parental psychiatric disorder, parental criminality, parental marital status, parental low socioeconomic status, and parental low educational achievement.
Early short-term = placed for the first time < age 13 and with < 1 year in total in out-of-home care (OHC) < age 18. Early intermediate = placed for the first time < age 13 and with 1–5 years in total in OHC < age 18. Long-term = placed for the first time < age 13 and with > 5 years in total in OHC < age 18. Teenage placement = placed for the first time ≥ age 13, regardless of total time in OHC < age 18.

**Table S6.**<br>Main and interaction effects of the Cox proportional hazard regression analysis of placement characteristic group, parental death, and substance-use disorders, stratified by sex.		
	Women (n = 459,712/48.70)	Men (n = 484,426/51.30)
Variables	Adjusted HR (95% CI)	Adjusted HR (95% CI)
Placement characteristic group		
Early short-term	1.69 (1.42, 2.01)	1.65(1.45, 1.88)
Early intermediate	2.67 (2.22, 3.21)	2.75 (2.42, 3.13)
Long-term	2.16 (1.85, 2.53)	2.06 (1.83, 2.32)
Teenage placement	6.38 (5.86, 6.95)	7.26 (6.82, 7.74)
Parental death	1.35 (1.17, 1.56)	1.32 (1.18, 1.47)
Interaction between placement characteristic group and parental death		
Early short-term × parental death	0.66 (0.31, 1.44)	0.82 (0.50, 1.36)
Early intermediate × parental death	0.62 (0.32, 1.21)	0.47 (0.25, 0.86)
Long-term × parental death	0.79 (0.42, 1.48)	0.92 (0.57, 1.48)
Teenage placement × parental death	0.53 (0.36, 0.79)	0.60 (0.45, 0.79)

Note. HR = hazard ratio, *CI* = confidence interval. The reference category included those participants without any record of substance-use disorder (women: n =452,274, men: n = 471,082). Reference for placement characteristic groups: no out-of-home care. Reference category parental death: no parental death. The adjusted model controlled for parental psychiatric disorder, parental criminality, parental marital status, parental low socioeconomic status, and parental low educational achievement.
Early short-term = placed for the first time < age 13 and with < 1 year in total in out-of-home care (OHC) < age 18. Early intermediate = placed for the first time < age 13 and with 1–5 years in total in OHC < age 18. Long-term = placed for the first time < age 13 and with > 5 years in total in OHC < age 18. Teenage placement = placed for the first time ≥ age 13, regardless of total time in OHC < age 18.

**Table S7.**<br>Main and interaction effects of the Cox proportional hazard regression analysis of placement characteristic group, parental death, and schizophrenia/schizotypal disorders, stratified by sex.		
	Women (n = 459,712/48.70)	Men (n = 484,426/51.30)
Variables	Adjusted HR (95% CI)	Adjusted HR (95% CI)
Placement characteristic group		
Early short-term	1.29 (0.91, 1.83)	1.68 (1.31, 2.15)
Early intermediate	1.26 (0.78, 2.06)	2.80 (2.18, 3.59)
Long-term	3.09 (2.37, 4.02)	2.64 (2.13, 3.27)
Teenage placement	4.40 (3.71, 5.21)	4.74 (4.14, 5.43)
Parental death	1.11 (0.85, 1.44)	1.28 (1.05, 1.56)
Interaction between placement characteristic group and parental death		
Early short-term × parental death	n/e	0.38 (0.09, 1.56)
Early intermediate × parental death	0.56 (0.07, 4.26)	0.18 (0.02, 1.31)
Long-term × parental death	0.49 (0.12, 2.06)	0.46 (0.14, 1.48)
Teenage placement × parental death	0.84 (0.39, 1.79)	0.76 (0.43, 1.35)

Note. HR = hazard ratio, *CI* = confidence interval. N/E = not estimable. The estimates for some interaction terms were not estimable due to low event counts in specific strata. The reference category included those participants without any record of schizophrenia/schizotypal disorder (women: n = 456,985, men: n = 480,451). Reference for placement characteristic groups: no out-of-home care. Reference category parental death: no parental death. The adjusted model controlled for parental psychiatric disorder, parental criminality, parental marital status, parental low socioeconomic status, and parental low educational achievement.

Early short-term = placed for the first time < age 13 and with < 1 year in total in out-of-home care (OHC) < age 18. Early intermediate = placed for the first time < age 13 and with 1–5 years in total in OHC < age 18. Long-term = placed for the first time < age 13 and with > 5 years in total in OHC < age 18. Teenage placement = placed for the first time ≥ age 13, regardless of total time in OHC < age 18.

Table S8.
Main and interaction effects of the Cox proportional hazard regression analysis of placement characteristic group, parental death, and mood/affective disorders, stratified by sex.

	Women (*n* = 459,712/48.70)	Men (*n* = 484,426/51.30)
Variables	Adjusted HR (95% CI)	Adjusted HR (95% CI)
Placement characteristic group		
Early short-term	1.55 (1.30, 1.84)	1.25 (1.00, 1.55)
Early intermediate	1.66 (1.33, 2.08)	1.58 (1.24, 2.01)
Long-term	1.95 (1.65, 2.30)	1.31 (1.07, 1.62)
Teenage placement	3.45 (3.11, 3.81)	2.87 (2.52, 3.26)
Parental death	1.30 (1.14, 1.48)	1.34 (1.16, 1.55)
Interaction between placement characteristic group and parental death		
Early short-term × parental death	0.42 (0.15, 1.15)	0.56 (0.20, 1.55)
Early intermediate × parental death	0.63 (0.27, 1.46)	0.16 (0.02, 1.13)
Long-term × parental death	0.71 (0.35, 1.47)	0.59 (0.22, 1.62)
Teenage placement × parental death	0.45 (0.26, 0.77)	0.72 (0.42, 1.24)

Note. HR = hazard ratio, *CI* = confidence interval. The reference category included those participants without any record of mood/affective disorder (women: *n* = 450,421, men: *n* = 477,550). Reference for placement characteristic groups: no out-of-home care. Reference category parental death: no parental death. The adjusted model controlled for parental psychiatric disorder, parental criminality, parental marital status, parental low socioeconomic status, and parental low educational achievement.
Early short-term = placed for the first time < age 13 and with < 1 year in total in out-of-home care (OHC) < age 18. Early intermediate = placed for the first time < age 13 and with 1–5 years in total in OHC < age 18. Long-term = placed for the first time < age 13 and with > 5 years in total in OHC < age 18. Teenage placement = placed for the first time ≥ age 13, regardless of total time in OHC < age 18.

Table S9.
Main and interaction effects of the Cox proportional hazard regression analysis of placement characteristic group, parental death, and anxiety/stress-related and somatoform disorders, stratified by sex.

	Women (*n* = 459,712/48.70)	Men (*n* = 484,426/51.30)
Variables	Adjusted HR (95% CI)	Adjusted HR (95% CI)
Placement characteristic group		
Early short-term	1.53 (1.29, 1.81)	1.66 (1.38, 1.98)
Early intermediate	1.88 (1.53, 2.31)	1.87 (1.52, 2.30)
Long-term	2.33 (2.01, 2.71)	1.92 (1.63, 2.26)
Teenage placement	4.66 (4.27, 5.09)	4.12 (3.71, 4.57)
Parental death	1.24 (1.09, 1.42)	1.30 (1.13, 1.50)
Interaction between placement characteristic group and parental death		
Early short-term × parental death	0.98 (0.49, 1.95)	0.28 (0.09, 0.89)
Early intermediate × parental death	0.54 (0.24, 1.25)	1.23 (0.63, 2.40)
Long-term × parental death	0.59 (0.29, 1.21)	0.54 (0.24, 1.25)
Teenage placement × parental death	0.47 (0.29, 0.75)	0.42 (0.24, 0.73)

Note. HR = hazard ratio, *CI* = confidence interval. The reference category included those participants without any record of anxiety/stress-related and somatoform disorder (women: *n* = 449,839, men: *n* = 476,964). Reference for placement characteristic groups: no out-of-home care. Reference category parental death: no parental death. The adjusted model controlled for parental psychiatric disorder, parental criminality, parental marital status, parental low socioeconomic status, and parental low educational achievement.
Early short-term = placed for the first time < age 13 and with < 1 year in total in out-of-home care (OHC) < age 18. Early intermediate = placed for the first time < age 13 and with 1–5 years in total in OHC < age 18. Long-term = placed for the first time < age 13 and with > 5 years in total in OHC < age 18. Teenage placement = placed for the first time ≥ age 13, regardless of total time in OHC < age 18.

Table S10.
Main and interaction effects of the Cox proportional hazard regression analysis of placement characteristic group, parental death, and behavioral/physiological syndromes, stratified by sex.

	Women (*n* = 459,712/48.70)	Men (*n* = 484,426/51.30)
Variables	Adjusted HR (95% CI)	Adjusted HR (95% CI)
Placement characteristic group		
Early short-term	1.50 (0.96, 2.35)	1.97 (0.92, 4.25)
Early intermediate	1.74 (0.97, 3.10)	2.20 (0.89, 5.46)
Long-term	1.97 (1.26, 3.06)	3.31 (1.76, 6.22)
Teenage placement	3.68 (2.91, 4.66)	2.88 (1.68, 4.94)
Parental death	0.89 (0.63, 1.27)	0.97 (0.48, 1.97)
Interaction between placement characteristic group and parental death		
Early short-term × parental death	n/e	n/e
Early intermediate × parental death	0.98 (0.12, 7.74)	n/e
Long-term × parental death	n/e	1.72 (0.20, 14.93)
Teenage placement × parental death	0.50 (0.12, 2.13)	2.36 (0.46, 12.09)

Note. HR = hazard ratio, *CI* = confidence interval. N/E = not estimable. The estimates for some interaction terms were not estimable due to low event counts in specific strata. The reference category included those participants without any record of behavioral/physiological syndrome (women: *n* = 457,785, men: *n* = 484,002). Reference for placement characteristic groups: no out-of-home care. Reference category parental death: no parental death. The adjusted model controlled for parental psychiatric disorder, parental criminality, parental marital status, parental low socioeconomic status, and parental low educational achievement.
Early short-term = placed for the first time < age 13 and with < 1 year in total in out-of-home care (OHC) < age 18. Early intermediate = placed for the first time < age 13 and with 1–5 years in total in OHC < age 18. Long-term = placed for the first time < age 13 and with > 5 years in total in OHC < age 18. Teenage placement = placed for the first time ≥ age 13, regardless of total time in OHC < age 18.

Table S11.
Main and interaction effects of the Cox proportional hazard regression analysis of placement characteristic group, parental death, and personality disorders, stratified by sex.

	Women (*n* = 459,712/48.70)	Men (*n* = 484,426/51.30)
Variables	Adjusted HR (95% CI)	Adjusted HR (95% CI)
Placement characteristic group		
Early short-term	1.90 (1.38, 2.60)	2.92 (2.14, 3.99),
Early intermediate	3.11 (2.22, 4.36)	4.86 (3.59, 6.59)
Long-term	4.53 (3.58, 5.72)	3.60 (2.72, 4.78)
Teenage placement	9.45 (8.27, 10.80)	9.49 (8.02, 11.22)

Parental death	1.54 (1.21, 1.95)	1.30 (0.93, 1.82)
Interaction between placement characteristic group and parental death		
Early short-term × parental death	0.30 (0.04, 2.20)	n/e
Early intermediate × parental death	0.19 (0.03, 1.37)	0.23 (0.03, 1.73)
Long-term × parental death	0.39 (0.12, 1.28)	0.24 (0.03, 1.81)
Teenage placement × parental death	0.58 (0.33, 1.02)	0.38 (0.16, 0.91)

Note. HR = hazard ratio, *CI* = confidence interval. N/E = not estimable. The estimates for some interaction terms were not estimable due to low event counts in specific strata. The reference category included those participants without any record of personality disorder (women: n = 457,068, men: n = 482,892). Reference for placement characteristic groups: no out-of-home care. Reference category parental death: no parental death. The adjusted model controlled for parental psychiatric disorder, parental criminality, parental marital status, parental low socioeconomic status, and parental low educational achievement.
Early short-term = placed for the first time < age 13 and with < 1 year in total in out-of-home care (OHC) < age 18. Early intermediate = placed for the first time < age 13 and with 1–5 years in total in OHC < age 18. Long-term = placed for the first time < age 13 and with > 5 years in total in OHC < age 18. Teenage placement = placed for the first time ≥ age 13, regardless of total time in OHC < age 18.
